# Onset of static and dynamic universality among molecular models of polymers

**DOI:** 10.1038/s41598-017-08501-0

**Published:** 2017-09-28

**Authors:** Kazuaki Z. Takahashi, Ryuto Nishimura, Nobuyoshi Yamato, Kenji Yasuoka, Yuichi Masubuchi

**Affiliations:** 1 Multi-scale Soft-matter Simulation Team, Research Center for Computational Design of Advanced Functional Materials, National Institute of Advanced Industrial Science and Technology (AIST), Central 2, 1-1-1 Umezono, Tsukuba, Ibaraki 305-8568 Japan; 2 Department of Mechanical Engineering, Keio University, 3-14-1 Hiyoshi, Kohoku-ku, Yokohama, Kanagawa 223-8522 Japan; 3 National Composite Center, Nagoya University, Furocho, Chikusa, Nagoya 464-8630 Japan

## Abstract

A quantitatively accurate prediction of properties for entangled polymers is a long-standing challenge that must be addressed to enable efficient development of these materials. The complex nature of polymers is the fundamental origin of this challenge. Specifically, the chemistry, structure, and dynamics at the atomistic scale affect properties at the meso and macro scales. Therefore, quantitative predictions must start from atomistic molecular dynamics (AMD) simulations. Combined use of atomistic and coarse-grained (CG) models is a promising approach to estimate long-timescale behavior of entangled polymers. However, a systematic coarse-graining is still to be done for bridging the gap of length and time scales while retaining atomistic characteristics. Here we examine the gaps among models, using a generic mapping scheme based on power laws that are closely related to universality in polymer structure and dynamics. The scheme reveals the characteristic length and time for the onset of universality between the vastly different scales of an atomistic model of polyethylene and the bead-spring Kremer–Grest (KG) model. The mapping between CG model of polystyrene and the KG model demonstrates the fast onset of universality, and polymer dynamics up to the subsecond time scale are observed. Thus, quantitatively traceable timescales of polymer MD simulations can be significantly increased.

## Introduction

Entangled polymers are widely used in many industrial applications. Despite many years of basic polymer research and industrial use, quantitatively accurate predictions of polymer properties is a long-standing problem that is of great importance for efficient improvement of those properties. The complex structures, dynamics, and physical properties of polymers can change dramatically over different time scales^1–3^. However, phenomena occurring over a wide range of timescales are closely related to each other; *i.e*., parameters at the atomistic scale can affect properties at the meso and macro scales. Therefore, predictions of polymer properties require atomistic molecular dynamics (AMD) simulations.

While recent advances in computation have enabled a wide range of AMD simulations of chemical and biological polymers^4–12^, fully atomistic simulations of entangled polymer dynamics over long timescales are beyond the capability of current platforms. Thus, coarse-grained (CG) MD simulations have been used for long timescales. They can accelerate simulations by reducing the number of degrees of freedom and increasing the fundamental time scale. Accessible polymer characteristics depend on the coarse-graining level, which determines the smallest length scale to trace the polymer dynamics. Models with various CG levels have been developed for long timescale behaviors of polymers^13–18^, and some of the models have great potential for more widespread use. However, one critical issue remains unresolved: namely, systematically determining the CG level to which polymers can be coarse-grained while still appropriately tracing static and dynamic polymer properties^18–22^. One path to attack this challenge is the direct comparison of polymer properties between atomistic and CG models. All CG models can be quantitatively compared with atomistic models through a generic mapping scheme based on experimentally established universality in the structures and dynamics of polymers having different chemistries^1,2,21,23–29^. Here, we examined the gap of length and time scales among models using the mapping scheme that assumed universality for three properties closely related to polymer dynamics that have been described in the Rouse theory^30^. These are the mean square end-to-end distance, the end-to-end relaxation time, and the diffusion coefficient. The gaps were estimated by mapping between the atomistic model of polyethylene (PE) and the bead-spring Kremer–Grest (KG) model^14,17^, a linkage between the vastly different scales of AMD and high-level CGMD. The gaps were also estimated by mapping between the multiscale molecular model of polystyrene (PS)^31^ and the KG model, which involves linkage among the three different scales AMD, middle-level CGMD, and high-level CGMD. PE and PS are common commercial polymers that have been extensively studied experimentally and theoretically, and several reliable molecular models are currently available. The KG model is the simplest very high-level CG model; it has been widely used for decades. With the exception of plain molecular geometries and excluded volume effects, chemical details are entirely omitted in the KG model. Despite its simplicity, it is highly useful and has been used in a wide range of studies on polymer nanocomposites^32,33^, polymer welding^34^, polymer brushes^35–37^, poly-electrolyte gels^38^, thermoresponsive polymers^39^, ring polymers^40^, polymer collapse^41^, healing of polymer interfaces^42,43^, and biopolymeric motions^44^. With using the generic mapping scheme, the spatial and temporal gaps among molecular models are systematically and accurately estimated. The gaps provide useful information for determination of appropriate CG level, showing a significant advance that will enable a quantitative solution for the challenging problems described above.

## Methodology

For quantitative comparison among polymer models, the length and time scales of CG models should be rescaled by a generic way. Because of the Gaussian nature of long polymers, the length unit is usually arbitrary when the linkage to atomistic models is attempted for the static properties of the global polymer structure. Therefore, we assume universality for the onset of entanglement. The onset specifies the characteristic molecular weight at which the power law exponents describing the relationships between dynamical parameters and the molecular weight change significantly. If this universality is assumed, the length units of two different polymer models can be linked to each other. Once the length unit is fixed, the unit of time can be determined. In CG models, it is determined by comparing dynamical measurements with those obtained by the corresponding atomistic model. For example, the mean square displacement (MSD) of chain centers has been used^22,31,45–51^, based on reptation theory that predicts the inflection point in MSD at the characteristic time of entanglement. This strategy is useful because the characteristic time of entanglement does not depend on the molecular weight. Despite the successful use of the linkage strategy, the universality of static and dynamic polymer properties is not fully attained for molecular models used in polymer simulations. Gaussian statistics assumed in Rouse models are not observed in atomistic models unless the molecular weight is sufficiently high. The non-Gaussian nature affects the dynamics, which deviate from predictions of the Rouse model. While these deviations are often concealed in the power law expressions^52,53^, this issue should be carefully considered when linking different models.

To obtain a reasonable linkage between the atomistic and CG models, we performed the following four steps. (i) The power law relations between the molecular weight *M* and the mean square end-to-end distance 〈*R* ^2^〉, the end-to-end relaxation time *τ*_R_, and the diffusion coefficient *D*, were evaluated for the atomistic and CG models (see Supplement Material for the simulation conditions). (ii) To determine the mass scaling factor, the critical molecular weight *M*_c_ was estimated from the change in *τ*_R_ – *M* and *D* – *M* power law exponents that indicate the onset of entanglement. The entanglement molecular weight, *M*_e_, was also estimated by primitive pass analysis (PPA)^54,55^ (see Supplement Material for the results of PPA). (iii) The spatial and temporal scaling factors were calculated using a set of 〈*R* ^2^〉, *τ*_R_, and *D* for each *M*, under the conditions of *M* ≤ *M*_c_. (iv) The validity of the scaling factors was evaluated by comparison of 〈*R* ^2^〉–*M*, *τ*_R_–*M*, and *D* – *M* power law relations obtained from AMD with those obtained from CGMD, and rescaled by the mass, length, and time scaling factors. Based on these results, a simple and reasonable mapping scheme was evaluated.

## Results and Discussion

### Evaluation of mapping scheme

Table 1 lists the representative parameters used for the estimation of scaling factors. The variables *m*, *σ*, and *τ* are the units of mass, length, and time for KGMD, respectively. The mass scaling factor can be accurately determined from the *M*_*e*_ or *M*_C_ results (for more details of the *M*_e_ or *M*_C_ values, see Supplement Material for the results of PPA). The spatial and temporal scaling factors can be determined by using any two of the following property combinations: 〈*R* ^2^〉_A*MD*_/〈*R* ^2^〉_K*GMD*_, *τ*_R,*AMD*_/*τ*_R,*KGMD*_, and D_A*MD*_/D_K*GMD*_ (suffixes “AMD” and “KGMD” denote the type of simulation). Therefore, three sets of two factors can be obtained from each condition of *M*. For the spatial scaling factor, the largest differences between the sets were 19%, 11%, and 2.2% for the smallest *M*, *M*_*e*_, and *M*_c_ values, respectively. For the temporal scaling factor, the largest differences between the sets were 43%, 23%, and 4.5% for the smallest *M*, *M*_e_, and *M*_c_ values, respectively. Thus, the scaling factors converge at *M* = *M*_c_, irrespective of the particular combination of these three properties. The results also imply that the AMD and KGMD simulations will be in quantitative agreement for the three power laws mentioned above. Therefore, the following equations can be used for estimating the spatial and temporal scaling factors,

1 $$1\,\sigma ={({\langle {R}^{2}\rangle }_{{\rm{c}},AMD}/{\langle {R}^{2}\rangle }_{{\rm{c}},KGMD})}^{\mathrm{1/2}}[{\rm{n}}m],$$

2 $$1\,\tau ={\tau }_{{\rm{c}},AMD}/{\tau }_{{\rm{c}},KGMD}[{\rm{p}}s],$$

where 〈*R* ^2^〉_c_ and *τ*_c_ are 〈*R* ^2^〉 and *τ*_R_ at *M* = *M*_c_, respectively. Note that Eqs (1) and (2) are avaliable for any two different MD simulations. From Table 1, it is clear that *D*_A*MD*_/*D*_K*GMD*_ should not be used to estimate the scaling factors because the standard errors or deviations were higher for *D* than for 〈*R* ^2^〉 and *τ*_R_.

**Table 1:** Representative parameters for estimation of scaling factors.

*M* conditions	AMD (standard error)				KGMD (standard error)			
	*M* [g/mol]	〈*R* ^2^〉 [n*m* ^2^]	*τ*_R_ [p*s*]	*D* [10^−3^ n*m* ^2^/ps]	*M* [*m*]	〈*R* ^2^〉 [*σ* ^2^]	*τ*_R_ [*τ*]	*D* [10^−3^ *σ* ^2^/*τ*]
Smallest	282.5	2.61962 (0.00087)	41.19 (0.22)	4.50 (0.17)	20	29.409 (0.020)	414.8 (4.5)	3.51 (0.26)
≈M_e_	703.4	8.5151 (0.0098)	266.2 (2.1)	1.473 (0.060)	50	80.42 (0.19)	3108 (49)	0.967 (0.078)
*M*_c_	983.9	12.515 (0.021)	552.1 (5.2)	0.914 (0.039)	70	114.72 (0.39)	6910 (130)	0.640 (0.055)

**Figure 1:**
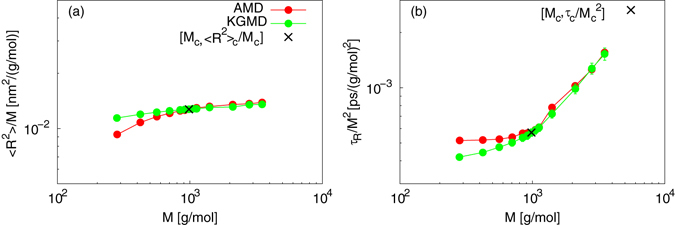
Comparison of power laws between AMD of PE and rescaled KGMD. (**a**) 〈*R* ^2^〉 – *M* power law. (**b**) *τ*_R_ – *M* power law.

The above scaling factors should be evaluated by comparing 〈*R* ^2^〉 – *M*, *τ*_R_ – *M*, and *D* – *M* power laws from AMD simulations with those obtained from the rescaled KGMD data. Figure 1(a) plots the 〈*R* ^2^〉 – *M* power law. The rescaled KGMD results begin to coincide with those from AMD at *M* = *M*_e_, and are almost equal to the AMD values at *M* ≥ *M*_c_. This indicates that the spatial scaling factor is reasonable. In contrast, a discrepancy between AMD and KGMD is observed at *M* < *M*_e_. This implies that the spatial scaling factors calculated at *M* < *M*_e_ are inadequate for quantitative static mapping. Figure 1(b) plots the *τ*_R_ – *M* power law. Similar to the 〈*R* ^2^〉 results, those from rescaled KGMD start to coincide with the AMD data at *M* = *M*_e_ and are almost identical to AMD values at *M* ≥ *M*_c_. This indicates that the temporal scaling factor is reasonable. In contrast, a disagreement between AMD and KGMD was seen at *M* < *M*_e_. This implies that the temporal scaling factors calculated at *M* < *M*_e_ are inadequate for quantitative dynamic mapping. For the *D* – *M* power law, the rescaled KGMD results are almost identical to the AMD data (see Fig. S1). Despite the discrepancies between the two types of simulations at *M* < *M*_e_ for 〈*R* ^2^〉 and *τ*_R_ (even at *M* < *M*_e_), the results of KGMD were close to the AMD results. This implies that the use of *D* may not lead to an accurate estimation of the scaling factors.

These results suggest a simple and reasonable mapping scheme consisting of three steps. (i) Estimate *M*_c_ for atomistic and CG models from *τ*_R_ – *M* (and *D* – *M*) power law. (ii) Determine the spatial and temporal scaling factors using Eqs (1) and (2). (iii) Evaluate the accuracy of the factors by comparing the 〈*R* ^2^〉 – *M*, *τ*_R_ – *M*, and *D* – *M* power laws obtained from AMD with those obtained from rescaled CGMD. Note that the accuracy of the above scheme relies on the accuracy of the power laws. The uncertainty of power laws reported in previous studies^56–58^ implies that a lot of data for long-time trajectories are required for the accurate estimation of the laws. Therefore we carried out long-time MD simulations of atomistic PE (up to 1 microseconds) and bead-spring KG (up to 1 billion time steps) models for a number of initial structures (see Supplement Material for the simulation conditions).

### Mapping between atomistic PE and KG models

**Figure 2:**
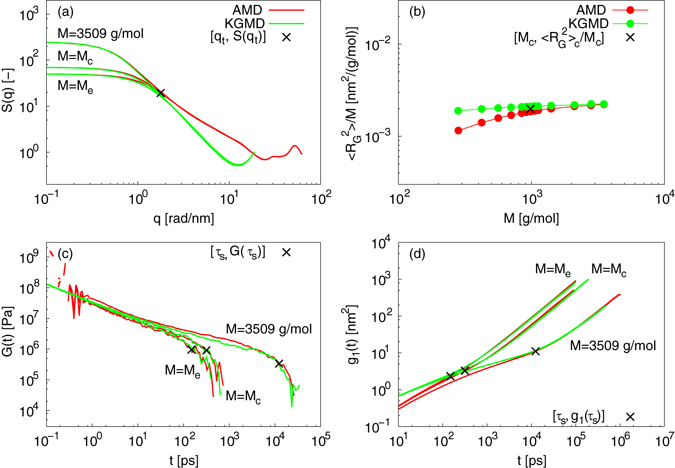
Results of mapping between AMD of PE and KGMD. (**a**) Static structure factors for *M* ≥ *M*_e_. (**b**) $\langle {R}_{{\rm{G}}}^{2}\rangle -M$ power law. (**c**) Relaxation modulus for *M* ≥ *M*_e_. Also plotted are [*τ*_s_,*G*(*τ*_s_)], where *τ*_s_, is a time at which a shoulder of *G*(*t*) occurs. (**d**) MSD of central monomers for *M* ≥ *M*_e_. Also plotted are [*τ*_s_,*g*_1_(*τ*_s_)], that correspond to the time at which the shoulder of *G*(*t*) occurs.

With using the present mapping scheme, the spacial and temporal gaps of representative static and dynamic properties can be accurately estimated. Figure 2(a) plots the static structure factor, (*S*(*q*), for *M* ≥ *M*_e_, where *q* is a spatial frequency that is equal to 2*π*/*r* (*r* is an intra- or intermolecular distance). The rescaled KGMD results begin to coincide with the AMD data at a threshold value *q*_t_ = 1.78 r*ad*/*nm*, indicating that the onset of static universality between AMD and KGMD occurs at a threshold length *r*_t_ = 3.5 n*m*, which is equal to the square root of 〈*R* ^2^〉_c_. This implies that the onset of static universality is closely related to *M*_c_. Note that the two simulations can be reasonably linked, even though a true plateau is not reached at the highest *M* of the present study. Figure 2(b) plots the power law relation between *M* and the radius of gyration, $\langle {R}_{{\rm{G}}}^{2}\rangle$. The rescaled KGMD results begin to coincide with the AMD values at *M* = *M*_c_. In contrast, the discrepancy between AMD and KGMD is observed at *M* < *M*_c_ because the onset of static universality occurs at *M* = *M*_c_, in agreement with the *S*(*q*) results. The results for the radial distribution function, *g*(*r*) (see Fig. S2), are consistent with those discussed above. Thus, we have demonstrated that our mapping scheme reveals the characteristic length for the onset of universality between the vastly different scales of AMD and KGMD, for the four static properties 〈*R* ^2^〉, $\langle {R}_{{\rm{G}}}^{2}\rangle$, *S*(*q*), and *g*(*r*). The spatial gap between AMD and KGMD is considerably large, raising the discrepancy at *M* < *M*_c_.

Figure 2(c) plots the relaxation modulus, *G*(*t*), for *M* ≥ *M*_e_, where *t* is time. We also plotted [*τ*_s_,*G*(*τ*_s_)], where *τ*_s_ is the time when the shoulder of *G*(*t*) occurs. For *M* = *M*_e_, *M*_c_, and 3509 g/mol, and *τ*_s_ = 149, 316, and 12400 p*s*, respectively. It was confirmed that *τ*_s_ is roughly proportional to *τ*_R_ and the terminal relaxation time *τ*_d_ (see Fig. S3). The rescaled KGMD results coincide with the AMD values at *t* ≥ *τ*_s_. The coincidence of the AMD and KGMD results at *t* < *τ*_s_ is partially observed. Figure 2(d) plots the MSD of central monomers, *g*_1_(*t*), for *M* ≥ *M*_e_. Also plotted are [*τ*_s_,*g*_1_(*τ*_s_)], for times corresponding to the shoulder of *G*(*t*). The rescaled KGMD results coincide with AMD values at *t* ≥ *τ*_s_, in contrast to the large discrepancy between AMD and KGMD data at *t* < *τ*_s_. This indicates that the onset of dynamic universality for AMD and KGMD occurs at *t* = *τ*_s_, and that a delay occurs in the onset of dynamic universality depending on *M*, because *τ*_s_ (which is roughly proportional to *τ*_R_ and *τ*_d_) increases exponentially with increasing *M*. This delay arises from differences in chemical structure between the two simulation methods. In KGMD, the chemical details are absent, whereas in AMD, they are included explicitly. This leads to differences in the details of the modeled entanglement. Effects of these differences completely disappear at *t* ≥ *τ*_s_. For the large *M* (=3509 g/mol), the coincidence of the AMD and KGMD results at *t* < *τ*_s_ is partially observed. This indicate that the delay in the onset of dynamic universality has a tendency to saturate with increasing *M*. The results of the autocorrelation function for the end-to-end vector *C*(*t*) were consistent with those for *τ*_R_ (see Fig. S4). Thus, the mapping scheme reveals the characteristic time for the onset of universality between the vastly different scales of AMD and KGMD, for the five dynamic properties *τ*_R_, *D*, *C*(*t*), *G*(*t*), and *g*_1_(*t*). The temporal gap between AMD and KGMD is considerably large, raising the discrepancy at *t* < *τ*_s_.

### Mapping between multiscale PS and KG models

**Figure 3:**
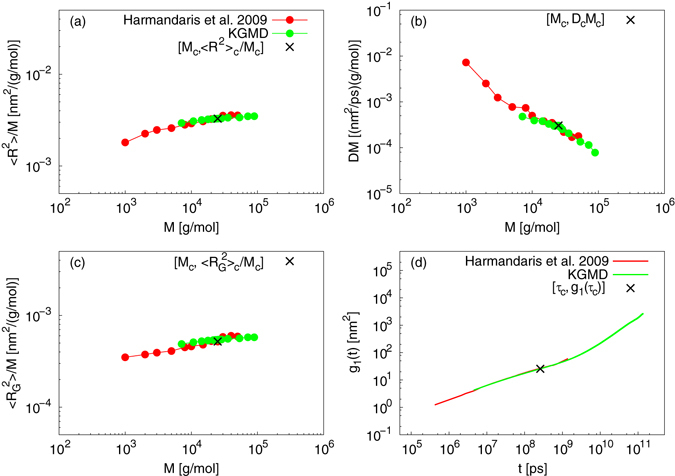
Results of mapping between MSMD of PS and KGMD. (**a**) 〈*R* ^2^〉 – *M* power law. (**b**) *D* – *M* power law. (**c**) $\langle {R}_{{\rm{G}}}^{2}\rangle -M$ power law. (**d**) MSD of central monomers at *M* = 50000 g/mol.

Harmandaris *et al*. performed multiscale MD (MSMD) simulations of PS melts^31^. The simulations were a combination of AMD and middle-level CGMD that are linked with a specialized mapping scheme. Data for 〈*R* ^2^〉, $\langle {R}_{{\rm{G}}}^{2}\rangle$, *D*, and *g*_1_(*t*) indicate that the rescaled CGMD provides quantitatively accurate results when compared to AMD and experimental data. Here, our mapping scheme was applied to estimate the gap between MSMD data (middle-level CGMD data rescaled by AMD data of PS) and that of KGMD. From the *D* – *M* power law of MSMD, *M*_c_,_*MSMD*_ = 25000 g/mol and 〈*R* ^2^〉_c_,_*MSMD*_ = 82.0 n*m* ^2^ (the suffix “MSMD” denotes MSMD simulation). The value *τ*_c,*MSMD*_ = 2.59 × 10^8^ p*s* is estimated from the convergence of the scaling factors discussed above: *τ*_c,*MSMD*_/*τ*_c,*KGMD*_ = (〈*R* ^2^〉_c,*MSMD*_/〈*R* ^2^〉_c,*KGMD*_)(*D*_c,*MSMD*_/*D*_c,*KGMD*_)^−1^. The spatial and temporal scaling factors for this mapping were determined from the above values, KGMD values, and Eqs (1) and (2). Figure 3(a) and (b) plot the 〈*R* ^2^〉 – *M* and *D* – *M* power laws, respectively. The rescaled KGMD results are almost identical to the MSMD data, confirming the validity of the spatial and temporal scaling factors. Figure 3(c) plots the $\langle {R}_{{\rm{G}}}^{2}\rangle -M$ power law. The rescaled KGMD results are almost equal to those of MSMD, which indicates that the static mapping is reasonable. There was no discrepancy between two models, unlike the mapping between AMD and KGMD. Thus, the spatial gap between the middle-level CG and the KG model is smaller than that between the atomistic and KG models. Figure 3(d) plots the MSD of central monomers at *M* = 50000 g/mol. The interaction points per chain of atomistic, CG, and KG models are 7680 atoms (or 3840 united atoms), 960 particles, and 140 beads, respectively. This implies the efficient multiscale mapping among the three molecular models. The rescaled KGMD results are almost equal to those of MSMD, which indicates that the dynamic mapping is reasonable. Furthermore, the results for *g*_1_(*t*) are a quantitative estimation of polymer dynamics up to a subsecond time scale (0.135 sec). This highlights the great potential of multiscale mapping for polymer molecular dynamics with realistic time scales. A direct comparison between MD results and experimental data becomes much easier for polymer dynamics, and enables high-throughput screening in polymer development. There was no discrepancy between two models, unlike the mapping between AMD and KGMD. The onset of universality in *g*_1_(*t*) is much *faster* (*t* ≪ *τ*_c_) than that for mapping between AMD and KGMD. Thus, the temporal gap between the middle-level CG and the KG model is smaller than that between the atomistic and KG models.

Again we focus on the characteristic of the spatial and temporal gaps. The large gaps are observed between AMD and KGMD despite the number of united-atoms per PE chain is almost equal to that of beads per KG chain. In contrast, The gaps between middle-level CGMD and KGMD is small despite the number of CG particles per PS chain is about 7 times larger than that of beads per KG chain. This means that the difference of chemical details (e.g., intra- and intermolecular interactions) is more effective for the gaps than the difference of the plain geometries of polymer models. Significantly, the gaps become small when linking molecular models having not less than a certain CG level. Estimating this level is critical for the development of CG models, because it provides the threshold for the effective coarse-graining achieving a good balance between model accuracy and computational cost. Here the present mapping scheme can reasonably determine the spatial and temporal gaps for representative polymer properties. The quantified gaps are useful as the index for searching the appropriate CG level. Therefore, optimization techniques of coarse-graining for polymer molecular models will be systematically extended by using the present scheme.

## Conclusions

The application of universal polymer behavior to the linkage among molecular models at various length and time scales was examined in terms of a mapping scheme focused on power laws that also show universality. The spatial and temporal gaps were evaluated by mapping between the atomistic model of PE and the coarse-grained KG model, a linkage between the vastly different scales of AMD and high-level CGMD. The scheme reveals the characteristic length and time for the onset of universality between AMD and KGMD for the representative static and dynamic properties. The large gaps raise the discrepancies of the properties at short length and time scales, showing the clear limit for bridging the two simulations while retaining atomistic characteristics. To overcome this limit, low-level (*i.e*., elaborate) CG models that introduce the effects of short length and time scales should be developed^18,20,22^. The scheme was then use to evaluate the gaps between the multiscale molecular models of PS and the KG model, which involves a linkage among the three different scales of AMD, middle-level CGMD, and high-level CGMD. The small gaps between middle- and high-level CGMD attain the fast onset of universality without the discrepancies of the properties at any length and time scales. Furthermore, it allows a linkage between the two models that quantitatively estimates polymer dynamics up to the subsecond timescale. This demonstrates the great potential to that the combination of various scales of molecular models permits multiscale mapping for polymer MD simulations. Here, quantitatively traceable MD time ranges can be significantly scaled up, which introduces the possibility of direct linkage between MD simulations and macroscale approaches^59,60^. The present mapping scheme is simple, and the scaling factors can be estimated with reasonable accuracy by using existing MD simulations and computational resources. Therefore, it is anticipated that it will be applicable to the sequential multiscale mapping among AMD, low-, middle-, and high-level CGMD for quantitative estimation of polymer properties at realistic length and time scales while maintaining a reasonable computational cost.

## Electronic supplementary material

Supplementary Information
