# Supplementary material for: Onset of static and dynamic universality among molecular models of polymers
Source: Sci Rep. 2017 Sep 28;7:12379. doi: 10.1038/s41598-017-08501-0 (PMC5620073; doi:10.1038/s41598-017-08501-0)
Supplement: Supplementary file 1 — Supplementary Information [file 41598_2017_8501_MOESM1_ESM.pdf]

Supplement material for "Onset of static and dynamic universality among molecular models of polymers"

Kazuaki Z. Takahashi*

Multi-scale Soft-matter Simulation Team,

Research Center for Computational Design of Advanced Functional Materials,

National Institute of Advanced Industrial Science and Technology (AIST),

Central 2, 1-1-1 Umezono, Tsukuba, Ibaraki 305-8568, Japan

Ryuto Nishimura, Nobuyoshi Yamato, and Kenji Yasuoka

Department of Mechanical Engineering,

Keio University, 3-14-1 Hiyoshi, Kohoku-ku,

Yokohama, Kanagawa 223-8522, Japan

Yuichi Masubuchi

National Composite Center, Nagoya University,

Furocho, Chikusa, Nagoya 464-8630, Japan

(Dated: June 23, 2017)

* Department of Mechanical Engineering, Keio University, 3-14-1 Hiyoshi, Kohoku-ku, Yokohama, Kanagawa 223-8522, Japan; kazu.takahashi@aist.go.jp

SIMULATION CONDITIONS

We performed AMD simulations for PE melts using a united-atom model (TraPPE-UA[1]) and CGMD simulations for polymer melts using the KG model. United-atom PE systems studied in the present work are listed in Table S1. The term M varied from 282.5 g/mol to 3509 g/mol. Three to six different initial structures were used for each condition of M. Over hundreds of nanoseconds of time evolution in equilibrium state were traced for each initial structure. The simulations were performed with a constant particle number, volume, and temperature ensemble using the Nosé-Hoover thermostat[2, 3]. The density was 0.650 g/cm^3, and the temperature was 500 K. The Verlet leapfrog integrator[4] was used with three-dimensional periodic boundary conditions (PBCs) and a time step of 2 fs. The atoms in each PE molecule were constrained by the LINCS algorithm[5].

Coarse-grained KG model systems studied in the present work are listed in Table S2. The term M varied from 20 to 250 m. Three to six different initial structures were used for each condition of M. Over hundreds of millions of time steps in equilibrium state were traced for

TABLE S1. United-atom PE systems studied in the present work ($\rho = 0.65$ g/mol and $T = 500$ K)

M [g/mol]	No. of chains	Simulation time [ns]	No. of initial structures
282.556	1500	100	3
422.826	1000	100	3
563.096	750	100	3
703.366	600	100	3
843.636	500	100	3
913.771	461	100	3
983.906	428	100	3
1054.041	400	100	3
1124.176	375	100	3
1404.716	300	500	6
2106.066	200	500	6
2807.416	150	800	6
3508.766	120	1000	6

each initial structure. The simulations were performed using a constant particle number, volume, and temperature condition. The density was $0.85\,m/\sigma^3$, and the temperature was ε/k_B, where ε is the energy unit of KGMD, and k_B is Boltzmanns constant. The velocity Verlet integrator[6] was used with three-dimensional PBCs and a time step of $0.006\,\tau$. Terms m, σ, ε, and k_B were set to 1 in KGMD. The beads in each chain were constrained by the RATTLE algorithm[7]. All AMD and KGMD simulation systems were equilibrated prior to data acquisition.

TABLE S2. Coarse-grained KG model systems studied in the present work ($\rho = 0.85\,m/\sigma^3$ and $T = \varepsilon/k_\mathrm{B}$)

M [m]	No. of chains	Simulation time [τ]	No. of initial structures
20	1250	1.2×10^6	3
30	833	1.2×10^6	3
40	625	1.2×10^6	3
50	500	1.2×10^6	3
60	416	2.4×10^6	3
65	384	2.4×10^6	3
70	357	2.4×10^6	3
75	333	2.4×10^6	3
80	312	2.4×10^6	3
100	250	3.6×10^6	6
150	166	3.6×10^6	6
200	125	3.6×10^6	6
250	100	6.0×10^6	6

RESULTS OF PRIMITIVE PASS ANALYSIS

Table S3 shows the results of primitive pass analysis (PPA). PPA was performed using Z1 code[8–10] (http://www.complexfluids.ethz.ch/cgi-bin/Z1). AMD data at $M = 3509\,\mathrm{g/mol}$ and KGMD data at $M = 250\,m$ were used for PPA, respectively. The results of KGMD are almost the same as that of previous reports[8, 11], and the results of AMD correspond to that reported in Ref. [8]. The values of M_e were estimated using four different models called as the classical S-kink, modified S-kink, classical S-coil, and modified S-coil, respectively. Four different values of M_e were estimated from four different models. Importantly, the values $M_\mathrm{e,AMD}/M_\mathrm{e,KGMD}$ are almost same as the value $M_\mathrm{c,AMD}/M_\mathrm{c,KGMD} = 14.06\,(\mathrm{g/mol})/m$ (within 4.6 %), irrespective of the difference of the above models. This indicate that the mass scaling factor determined in this work is reasonable from the aspect of PPA.

As was mentioned by Hoy *et al.*, the value of M_e may not converge unless using data of sufficiently long polymer chains[8]. Thus we determine M_e from the comparison between data reported by Hoy *et al.* and that listed in Table S3, under the conditions of $M \leq M_\mathrm{c}$[12]. As the consequence, the results from the modified S-kink ($M_\mathrm{e,AMD} = 675.3\,\mathrm{g/mol}$ and $M_\mathrm{e,KGMD} = 48.55\,m$ in Table S3) are acceptable as the reasonable values. These values are very close to AMD at $M = 703.4\,\mathrm{g/mol}$ and KGMD at $M = 50\,m$, respectively (within 4.0 %). Therefore we choose the above conditions as $M \approx M_\mathrm{e}$ conditions. Note that the results of M_e and M_c do not conflict with the experimentally accepted relation $M_\mathrm{c}/M_\mathrm{e} \sim 2$, because it is also accepted that the relation is quite rough[12].

TABLE S3. Results of primitive pass analysis

Models	classical S-kink	modified S-kink	classical S-coil	modified S-coil
$M_\mathrm{e,AMD}$ [g/mol]	566.4	675.3	1061	1423
$M_\mathrm{e,KGMD}$ [m]	40.22	48.55	74.09	96.8
$M_\mathrm{e,AMD}/M_\mathrm{e,KGMD}$ [(g/mol)/m]	14.08	13.91	14.32	14.70

FIGURES

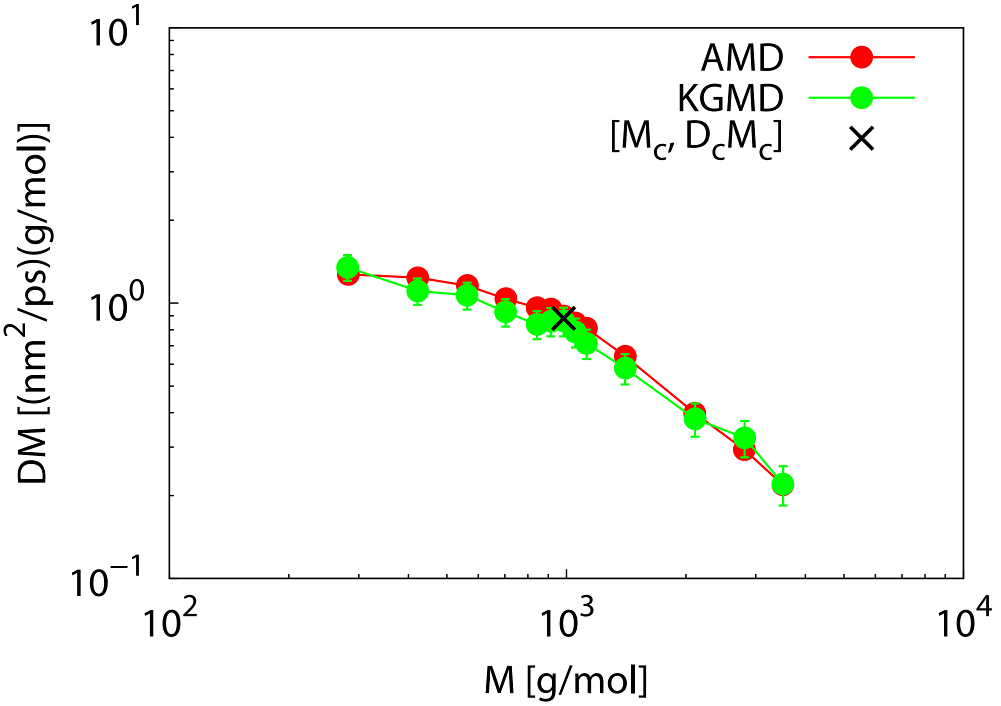

FIG. S1. Comparison of D–M power law between AMD of PE and rescaled KGMD.

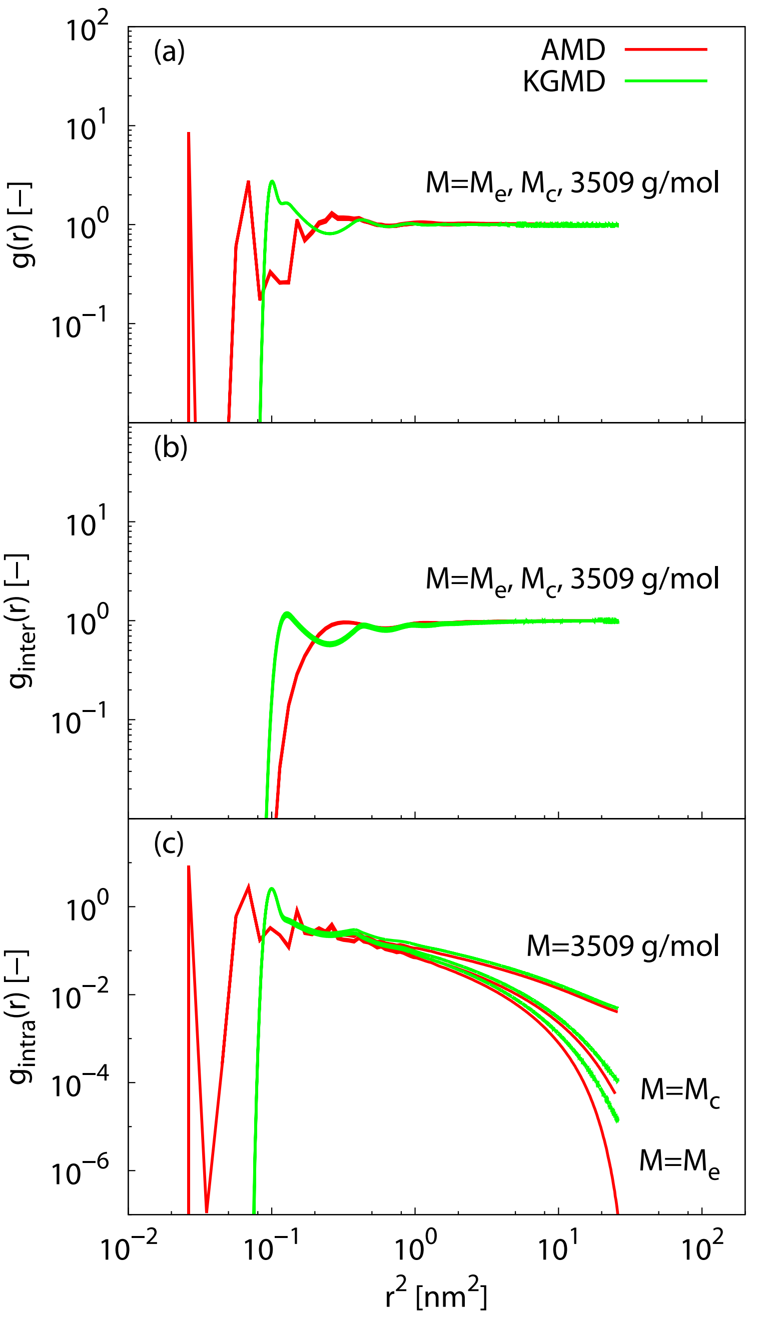

FIG. S2. Mapping results of $g(r)$ between AMD of PE and KGMD. (a) Total and (b) intermolecular contributions for $M \geq M_\mathrm{e}$. The rescaled KGMD results are almost equal to the AMD values at $r \sim 0.7\,\mathrm{nm} << r_\mathrm{t}$, irrespective of the M conditions. (c) Intramolecular contributions for $M \geq M_\mathrm{e}$. The rescaled KGMD results begin to coincide with the AMD values at $M = M_\mathrm{c}$. In contrast, the discrepancy between AMD and KGMD is observed at $M = M_\mathrm{e}$ because the onset of static universality occurs at $M = M_\mathrm{c}$, in agreement with the $S(q)$ and $\langle R_\mathrm{G}^2 \rangle$ results.

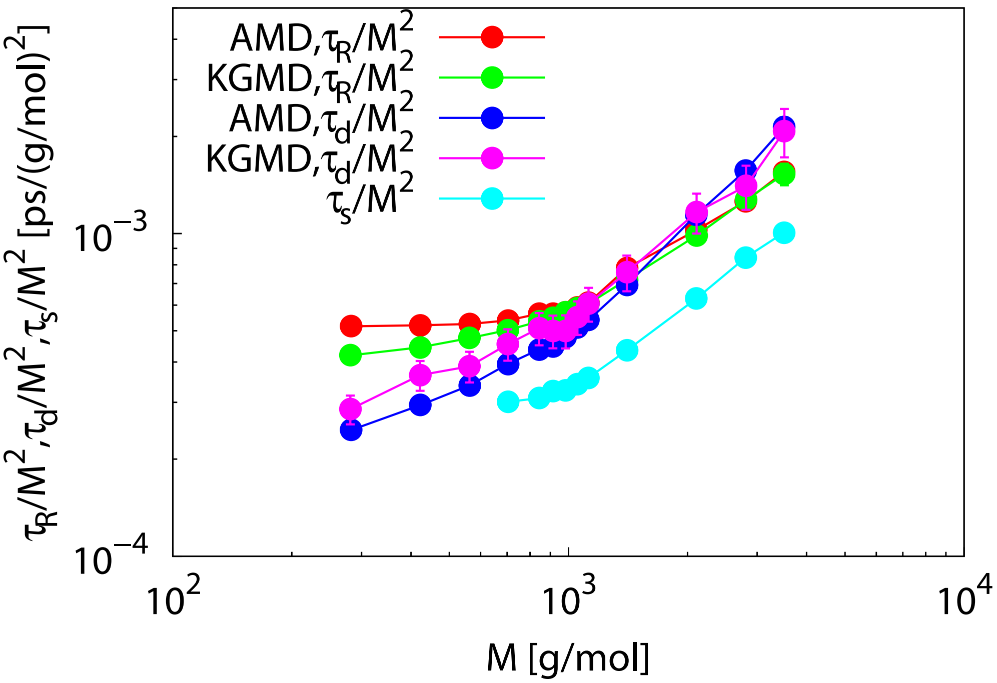

FIG. S3. Comparison of the τ_{R}–M, τ_{d}–M with τ_{s}–M power laws. The results of KGMD were rescaled. For $M \geq M_{\mathrm{e}}$, these are roughly proportional to each other, in the range of standard error for τ_{d} of rescaled KGMD. Note that τ_{d} was calculated using the relation $\tau_{\mathrm{d}} = \langle R^2 \rangle / 3\pi^2 D$[13].

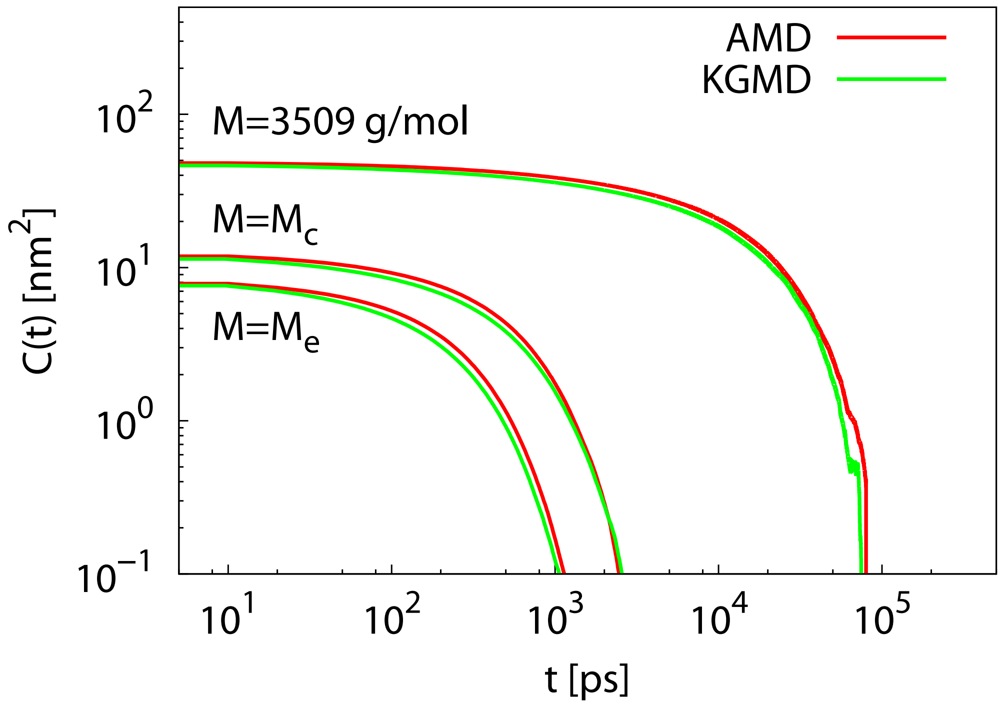

FIG. S4. Mapping results of $C(t)$ between AMD of PE and KGMD.

[1] M. G. Martin and J. I. Siepmann, The Journal of Physical Chemistry B **102**, 2569 (1998).

[2] W. G. Hoover, Physical Review A **31**, 1695 (1985).

[3] S. Nosé, The Journal of Chemical Physics **81**, 511 (1984).

[4] R. W. Hockney, Methods in Computational Physics **9**, 135 (1970).

[5] B. Hess, H. Bekker, H. J. Berendsen, J. G. Fraaije, *et al.*, Journal of computational chemistry **18**, 1463 (1997).

[6] W. C. Swope, H. C. Andersen, P. H. Berens, and K. R. Wilson, The Journal of Chemical Physics **76**, 637 (1982).

[7] H. Andersen, Journal of Computational Physics **52**, 24 (1983).

[8] R. S. Hoy, K. Foteinopoulou, and M. Kröger, Physical Review E **80**, 031803 (2009).

[9] M. Kröger, Computer Physics Communications **168**, 209 (2005).

[10] S. Shanbhag and M. Kröger, Macromolecules **40**, 2897 (2007).

[11] S. K. Sukumaran, G. S. Grest, K. Kremer, and R. Everaers, Journal of Polymer Science Part B: Polymer Physics **43**, 917 (2005).

[12] L. J. Fetters, D. J. Lohse, S. T. Milner, and W. W. Graessley, Macromolecules **32**, 6847 (1999).

[13] P. E. Rouse Jr, The Journal of Chemical Physics **21**, 1272 (1953).
